# Poly[[(μ_3_-5,6-dicarboxy­bicyclo­[2.2.2]oct-7-ene-2,3-dicarboxyl­ato)(1,10-phenanthroline)copper(II)] monohydrate]

**DOI:** 10.1107/S1600536808037999

**Published:** 2008-11-22

**Authors:** Yun-Yu Liu, Yu-Jiang Zhuo, Xing-Qi Li, Ji-Cheng Ma

**Affiliations:** a Department of Chemistry, Northeast Normal University, Changchun 130024, People’s Republic of China

## Abstract

In the title compound, {[Cu(C_12_H_10_O_8_)(C_12_H_8_N_2_)]·H_2_O}_*n*_, the Cu^II^ ion is five-coordinated by two N atoms from one phenanthroline ligand and three O atoms from three different H_2_ *L* ^2−^ anions (H_4_ *L* is bicyclo­[2.2.2]oct-7-ene-2,3,5,6-tetra­carboxylic acid) in a distorted square-pyramidal geometry. Each H_2_ *L* ^2−^ ion bridges three Cu^II^ atoms to form a zigzag sheet parallel to the *ab* plane. The crystal structure is consolidated by O—H⋯O hydrogen bonds.

## Related literature

For general background, see: Yang *et al.* (2008 ▶).
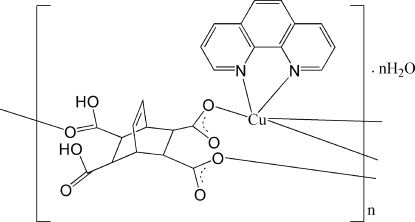


## Experimental

### 

#### Crystal data

[Cu(C_12_H_10_O_8_)(C_12_H_8_N_2_)]·H_2_O
*M* *_r_* = 543.96
Monoclinic, 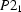

*a* = 6.5900 (4) Å
*b* = 15.1650 (8) Å
*c* = 10.7490 (6) Å
β = 95.244 (9)°
*V* = 1069.73 (10) Å^3^
*Z* = 2
Mo *K*α radiation
μ = 1.08 mm^−1^
*T* = 293 (2) K
0.33 × 0.21 × 0.20 mm

#### Data collection

Bruker APEX CCD area-detector diffractometer
Absorption correction: multi-scan (*SADABS*; Sheldrick, 1996 ▶) *T*_min_ = 0.696, *T*_max_ = 0.803
6580 measured reflections
4555 independent reflections
4343 reflections with *I* > 2σ(*I*)
*R*_int_ = 0.022

#### Refinement

*R*[*F* ^2^ > 2σ(*F* ^2^)] = 0.026
*wR*(*F* ^2^) = 0.065
*S* = 1.04
4555 reflections
333 parameters
2 restraints
H atoms treated by a mixture of independent and constrained refinement
Δρ_max_ = 0.31 e Å^−3^
Δρ_min_ = −0.37 e Å^−3^
Absolute structure: Flack (1983 ▶), 1914 Friedel pairs
Flack parameter: 0.008 (8)

### 

Data collection: *SMART* (Bruker, 1998 ▶); cell refinement: *SAINT* (Bruker, 1998 ▶); data reduction: *SAINT* (Bruker, 1998 ▶); program(s) used to solve structure: *SHELXS97* (Sheldrick, 2008 ▶); program(s) used to refine structure: *SHELXL97* (Sheldrick, 2008 ▶); molecular graphics: *SHELXTL-Plus* (Sheldrick, 2008 ▶); software used to prepare material for publication: *SHELXTL-Plus* (Sheldrick, 2008 ▶).

## Supplementary Material

Crystal structure: contains datablocks global, I. DOI: 10.1107/S1600536808037999/ci2718sup1.cif

Structure factors: contains datablocks I. DOI: 10.1107/S1600536808037999/ci2718Isup2.hkl

Additional supplementary materials:  crystallographic information; 3D view; checkCIF report

## Figures and Tables

**Table 1:** Selected bond lengths (Å)

N1—Cu1	2.0072 (17)
N2—Cu1	2.0119 (19)
O2—Cu1	1.9640 (15)
Cu1—O3^i^	1.9355 (15)
Cu1—O7^ii^	2.3398 (18)

Symmetry codes: (i) 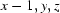; (ii) 
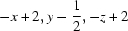
.

**Table 2:** Hydrogen-bond geometry (Å, °)

*D*—H⋯*A*	*D*—H	H⋯*A*	*D*⋯*A*	*D*—H⋯*A*
O5—H5⋯O1*W*	0.82	1.84	2.567 (2)	148
O8—H8⋯O2^iii^	0.82	1.79	2.594 (2)	166
O1*W*—H*W*11⋯O4^iv^	0.82 (3)	1.97 (3)	2.777 (3)	167 (3)
O1*W*—H*W*12⋯O1^v^	0.82 (2)	2.05 (3)	2.763 (3)	145 (4)

Symmetry codes: (iii) 
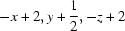
; (iv) 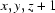; (v) 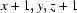.

**Fig. 1.**
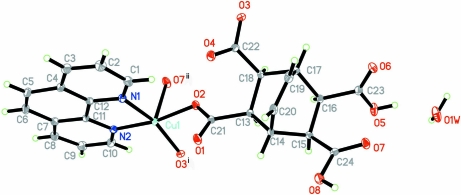
Part of the polymeric structure of the title compound, showing the atomic numbering scheme. Displacement ellipsoids are drawn at the 30% probability level. Symmetry codes: (i) x - 1, y, z; (ii) 2 - x, y - 1/2, 2 - z.

**Fig. 2.**
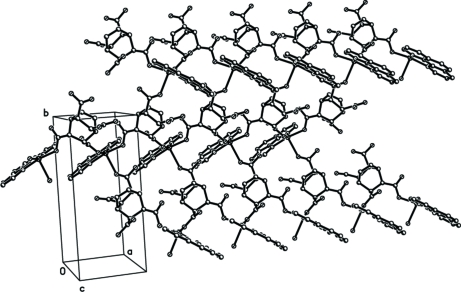
View of a zigzag sheet structure in the title compound.

## supplementary crystallographic information

### Comment

Coordination polymers based on poly(carboxylic acids) have been investigated in the area of solid state and material science (Yang *et al.*, 2008). We selected bicyclo[2.2.2]oct-7-ene-2,3,5,6-tetracarboxylic acid (H_4_L) as a poly(carboxylic acid) ligand and phenanthroline (phen) as a secondary ligand, generating a new coordination polymer, [Cu(phen)(H_2_L)]^.^H_2_O, which is reported here.

In the title compound, each Cu^II^ atom is five-coordinated by two N atoms from one phen ligand, and three O atoms from three different H_2_*L*^2-^ anions in a distorted square-pyramidal geometry (Fig. 1 and Table 1). Each H_2_*L*^2-^ bridges three Cu^II^ atoms to form a two-dimensional layer structure (Fig. 2). The O–H···O hydrogen bonds (Table 2) further consolidate the crystal structure.

### Experimental

A mixture of H_4_L (0.5 mmol), phen (0.5 mmol), NaOH (1 mmol) and CuCl_2_^.^2H_2_O (0.5 mmol) was suspended in deionized water (12 ml) and sealed in a 20-ml Teflon-lined autoclave. The mixture was heated at 373 K for 7 d and then the autoclave was slowly cooled to room temperature. The grown single crystals were collected, washed with deionized water and dried.

### Refinement

H atoms on C atoms were generated geometrically and refined as riding atoms with C—H = 0.93–0.98 Å and *U*_iso_(H) = 1.2*U*_eq_(C). The H atoms of the water molecules were located in a difference Fourier map and refined with an O—H distance restraint of 0.85 (1) Å and with *U*_iso_(H) = 1.2*U*_eq_(O).

### Crystal data

**Table:**

[Cu(C_12_H_10_O_8_)(C_12_H_8_N_2_)]·H_2_O	*F*_000_ = 558
*M**_r_* = 543.96	*D*_x_ = 1.689 Mg m^−^^3^
Monoclinic, *P*2_1_	Mo *K*α radiation λ = 0.71069 Å
Hall symbol: P 2yb	Cell parameters from 4555 reflections
*a* = 6.5900 (4) Å	θ = 1.1–28.4º
*b* = 15.1650 (8) Å	µ = 1.08 mm^−^^1^
*c* = 10.7490 (6) Å	*T* = 293 K
β = 95.244 (9)º	Block, blue
*V* = 1069.73 (10) Å^3^	0.33 × 0.21 × 0.20 mm
*Z* = 2	

### Data collection

**Table:**

Bruker APEX CCD area-detector diffractometer	4555 independent reflections
Radiation source: fine-focus sealed tube	4343 reflections with *I* > 2σ(*I*)
Monochromator: graphite	*R*_int_ = 0.022
*T* = 293 K	θ_max_ = 28.4º
φ and ω scans	θ_min_ = 2.3º
Absorption correction: multi-scan(SADABS; Sheldrick, 1996)	*h* = −8→8
*T*_min_ = 0.696, *T*_max_ = 0.803	*k* = −19→17
6580 measured reflections	*l* = −6→14

### Refinement

**Table:**

Refinement on *F*^2^	Hydrogen site location: inferred from neighbouring sites
Least-squares matrix: full	H atoms treated by a mixture of independent and constrained refinement
*R*[*F*^2^ > 2σ(*F*^2^)] = 0.026	*w* = 1/[σ^2^(*F*_o_^2^) + (0.0256*P*)^2^] where *P* = (*F*_o_^2^ + 2*F*_c_^2^)/3
*wR*(*F*^2^) = 0.065	(Δ/σ)_max_ = 0.001
*S* = 1.04	Δρ_max_ = 0.31 e Å^−^^3^
4555 reflections	Δρ_min_ = −0.37 e Å^−^^3^
333 parameters	Extinction correction: none
2 restraints	Absolute structure: Flack (1983), 1914 Friedel pairs
Primary atom site location: structure-invariant direct methods	Flack parameter: 0.008 (8)
Secondary atom site location: difference Fourier map	

### Special details

**Table:**

Geometry. All e.s.d.'s (except the e.s.d. in the dihedral angle between two l.s. planes) are estimated using the full covariance matrix. The cell e.s.d.'s are taken into account individually in the estimation of e.s.d.'s in distances, angles and torsion angles; correlations between e.s.d.'s in cell parameters are only used when they are defined by crystal symmetry. An approximate (isotropic) treatment of cell e.s.d.'s is used for estimating e.s.d.'s involving l.s. planes.
Refinement. Refinement of *F*^2^ against ALL reflections. The weighted *R*-factor *wR* and goodness of fit *S* are based on *F*^2^, conventional *R*-factors *R* are based on *F*, with *F* set to zero for negative *F*^2^. The threshold expression of *F*^2^ > σ(*F*^2^) is used only for calculating *R*-factors(gt) *etc*. and is not relevant to the choice of reflections for refinement. *R*-factors based on *F*^2^ are statistically about twice as large as those based on *F*, and *R*- factors based on ALL data will be even larger.

### Fractional atomic coordinates and isotropic or equivalent isotropic displacement parameters (Å^2^)

**Table:**

	*x*	*y*	*z*	*U*_iso_*/*U*_eq_	
C1	0.8692 (4)	0.81993 (17)	0.5050 (2)	0.0327 (5)	
H1	0.9536	0.8445	0.5700	0.039*	
C2	0.9222 (4)	0.82867 (19)	0.3826 (2)	0.0382 (6)	
H2	1.0408	0.8583	0.3669	0.046*	
C3	0.7992 (4)	0.79351 (17)	0.2862 (2)	0.0362 (6)	
H3	0.8341	0.7990	0.2047	0.043*	
C4	0.6199 (3)	0.74901 (17)	0.31010 (18)	0.0285 (5)	
C5	0.4749 (4)	0.71054 (18)	0.2161 (2)	0.0352 (6)	
H5A	0.4972	0.7156	0.1322	0.042*	
C6	0.3084 (4)	0.66765 (18)	0.2479 (2)	0.0361 (6)	
H6	0.2186	0.6434	0.1853	0.043*	
C7	0.2660 (4)	0.65841 (16)	0.3763 (2)	0.0291 (5)	
C8	0.1009 (4)	0.61105 (18)	0.4178 (2)	0.0364 (6)	
H8B	0.0083	0.5828	0.3606	0.044*	
C9	0.0779 (4)	0.60707 (19)	0.5428 (2)	0.0377 (6)	
H9	−0.0301	0.5758	0.5711	0.045*	
C10	0.2174 (4)	0.65023 (17)	0.6279 (2)	0.0329 (5)	
H10	0.1995	0.6473	0.7127	0.039*	
C11	0.4003 (3)	0.69806 (15)	0.46831 (19)	0.0240 (4)	
C12	0.5789 (3)	0.74318 (14)	0.43543 (18)	0.0237 (5)	
C13	1.0048 (3)	0.90548 (13)	0.94151 (18)	0.0192 (4)	
H13	0.9289	0.8814	1.0080	0.023*	
C14	1.0387 (3)	1.00356 (15)	0.97408 (18)	0.0223 (4)	
H14	0.9159	1.0382	0.9497	0.027*	
C15	1.0971 (3)	1.00950 (14)	1.11723 (18)	0.0220 (4)	
H15	0.9782	0.9919	1.1598	0.026*	
C16	1.2682 (3)	0.94109 (14)	1.14952 (18)	0.0211 (4)	
H16	1.1993	0.8873	1.1736	0.025*	
C17	1.3679 (3)	0.91758 (15)	1.02845 (19)	0.0229 (4)	
H17	1.4990	0.8879	1.0479	0.027*	
C18	1.2122 (3)	0.85576 (14)	0.95247 (18)	0.0203 (4)	
H18	1.1978	0.8014	1.0002	0.024*	
C19	1.3913 (3)	0.99816 (16)	0.94838 (19)	0.0288 (5)	
H19	1.5166	1.0168	0.9246	0.035*	
C20	1.2195 (4)	1.03987 (15)	0.91496 (19)	0.0268 (5)	
H20	1.2108	1.0873	0.8600	0.032*	
C21	0.8703 (3)	0.88961 (15)	0.82048 (19)	0.0208 (4)	
C22	1.2984 (3)	0.83259 (15)	0.82983 (19)	0.0237 (4)	
C23	1.4200 (4)	0.96369 (15)	1.2593 (2)	0.0265 (5)	
C24	1.1515 (3)	1.10347 (15)	1.1550 (2)	0.0240 (5)	
N1	0.7020 (3)	0.77771 (12)	0.53121 (15)	0.0253 (4)	
N2	0.3738 (3)	0.69509 (13)	0.59172 (16)	0.0251 (4)	
O1	0.7941 (3)	0.95074 (11)	0.75885 (15)	0.0331 (4)	
O2	0.8349 (2)	0.80701 (10)	0.79532 (13)	0.0256 (3)	
O1W	1.4570 (4)	0.98510 (18)	1.59374 (17)	0.0537 (6)	
O3	1.4401 (2)	0.77476 (12)	0.84210 (14)	0.0323 (4)	
O4	1.2381 (2)	0.86940 (12)	0.73066 (14)	0.0323 (4)	
O5	1.3290 (3)	0.95633 (14)	1.36461 (15)	0.0406 (4)	
H5	1.4105	0.9685	1.4244	0.061*	
O6	1.5975 (3)	0.97879 (13)	1.25429 (17)	0.0402 (4)	
O7	1.2819 (3)	1.12253 (12)	1.23587 (16)	0.0358 (4)	
O8	1.0345 (3)	1.16299 (13)	1.09436 (17)	0.0443 (5)	
H8	1.0703	1.2125	1.1181	0.066*	
Cu1	0.59195 (3)	0.760541 (17)	0.697564 (19)	0.02264 (7)	
HW11	1.378 (5)	0.952 (2)	1.625 (3)	0.050 (10)*	
HW12	1.575 (4)	0.969 (3)	1.613 (4)	0.078 (14)*	

### Atomic displacement parameters (Å^2^)

**Table:**

	*U*^11^	*U*^22^	*U*^33^	*U*^12^	*U*^13^	*U*^23^
C1	0.0300 (12)	0.0339 (14)	0.0336 (12)	−0.0050 (11)	0.0001 (10)	0.0004 (10)
C2	0.0364 (14)	0.0379 (15)	0.0413 (13)	−0.0056 (12)	0.0101 (11)	0.0070 (11)
C3	0.0433 (15)	0.0387 (14)	0.0276 (11)	0.0050 (11)	0.0095 (10)	0.0068 (9)
C4	0.0351 (11)	0.0263 (13)	0.0238 (9)	0.0057 (11)	0.0014 (8)	0.0009 (9)
C5	0.0486 (16)	0.0344 (15)	0.0218 (10)	0.0106 (11)	−0.0018 (10)	−0.0044 (9)
C6	0.0425 (15)	0.0343 (14)	0.0292 (11)	0.0029 (12)	−0.0098 (10)	−0.0086 (10)
C7	0.0311 (12)	0.0253 (12)	0.0300 (11)	0.0023 (10)	−0.0023 (9)	−0.0075 (9)
C8	0.0286 (12)	0.0344 (14)	0.0446 (14)	−0.0020 (11)	−0.0059 (10)	−0.0113 (10)
C9	0.0314 (13)	0.0350 (15)	0.0465 (14)	−0.0084 (11)	0.0030 (11)	−0.0058 (11)
C10	0.0318 (13)	0.0335 (14)	0.0339 (12)	−0.0055 (11)	0.0066 (10)	−0.0044 (10)
C11	0.0260 (11)	0.0210 (11)	0.0244 (10)	0.0044 (9)	−0.0014 (8)	−0.0037 (8)
C12	0.0270 (10)	0.0196 (13)	0.0240 (9)	0.0042 (8)	−0.0003 (8)	−0.0017 (7)
C13	0.0199 (10)	0.0184 (11)	0.0194 (9)	−0.0018 (8)	0.0014 (8)	−0.0009 (7)
C14	0.0236 (11)	0.0192 (10)	0.0229 (10)	0.0006 (9)	−0.0049 (8)	−0.0024 (8)
C15	0.0198 (10)	0.0224 (11)	0.0236 (10)	−0.0003 (8)	−0.0002 (8)	−0.0015 (8)
C16	0.0233 (10)	0.0187 (10)	0.0208 (9)	−0.0020 (8)	−0.0007 (8)	−0.0015 (7)
C17	0.0175 (10)	0.0259 (12)	0.0247 (10)	0.0002 (9)	−0.0007 (8)	−0.0037 (8)
C18	0.0199 (10)	0.0215 (11)	0.0190 (9)	0.0006 (8)	−0.0004 (7)	−0.0012 (7)
C19	0.0284 (12)	0.0312 (13)	0.0276 (11)	−0.0119 (10)	0.0062 (9)	−0.0042 (9)
C20	0.0357 (12)	0.0215 (11)	0.0223 (10)	−0.0076 (10)	−0.0026 (9)	0.0017 (8)
C21	0.0182 (10)	0.0220 (12)	0.0216 (10)	−0.0008 (9)	−0.0006 (8)	−0.0013 (8)
C22	0.0212 (10)	0.0254 (12)	0.0248 (10)	−0.0034 (8)	0.0036 (8)	−0.0029 (8)
C23	0.0330 (13)	0.0197 (11)	0.0256 (10)	0.0030 (9)	−0.0043 (9)	0.0010 (8)
C24	0.0238 (11)	0.0221 (12)	0.0262 (11)	0.0028 (9)	0.0031 (9)	−0.0032 (8)
N1	0.0252 (9)	0.0254 (12)	0.0251 (8)	−0.0010 (8)	0.0008 (7)	−0.0022 (7)
N2	0.0242 (9)	0.0245 (10)	0.0264 (9)	0.0010 (8)	0.0006 (7)	−0.0030 (7)
O1	0.0381 (10)	0.0263 (9)	0.0323 (9)	0.0023 (8)	−0.0117 (7)	0.0020 (7)
O2	0.0265 (8)	0.0205 (8)	0.0283 (7)	−0.0040 (7)	−0.0051 (6)	−0.0019 (6)
O1W	0.0514 (14)	0.0778 (17)	0.0291 (9)	−0.0253 (13)	−0.0121 (9)	0.0156 (10)
O3	0.0290 (8)	0.0397 (12)	0.0282 (7)	0.0106 (8)	0.0033 (6)	−0.0084 (7)
O4	0.0349 (9)	0.0392 (10)	0.0225 (7)	0.0009 (8)	0.0016 (6)	0.0024 (7)
O5	0.0447 (10)	0.0543 (13)	0.0211 (7)	−0.0153 (9)	−0.0054 (7)	−0.0004 (7)
O6	0.0254 (9)	0.0509 (13)	0.0424 (10)	0.0001 (8)	−0.0069 (7)	−0.0105 (8)
O7	0.0387 (10)	0.0224 (9)	0.0427 (10)	0.0004 (7)	−0.0164 (8)	−0.0049 (7)
O8	0.0502 (11)	0.0249 (10)	0.0528 (11)	0.0103 (9)	−0.0222 (9)	−0.0094 (8)
Cu1	0.02218 (12)	0.02490 (13)	0.02043 (10)	−0.00053 (12)	−0.00032 (8)	−0.00387 (11)

### Geometric parameters (Å, °)

**Table:**

C1—N1	1.326 (3)	C15—C16	1.548 (3)
C1—C2	1.398 (3)	C15—H15	0.98
C1—H1	0.93	C16—C23	1.515 (3)
C2—C3	1.364 (4)	C16—C17	1.552 (3)
C2—H2	0.93	C16—H16	0.98
C3—C4	1.405 (4)	C17—C19	1.511 (3)
C3—H3	0.93	C17—C18	1.564 (3)
C4—C12	1.401 (3)	C17—H17	0.98
C4—C5	1.448 (3)	C18—C22	1.523 (3)
C5—C6	1.346 (4)	C18—H18	0.98
C5—H5A	0.93	C19—C20	1.318 (3)
C6—C7	1.440 (3)	C19—H19	0.93
C6—H6	0.93	C20—H20	0.93
C7—C11	1.401 (3)	C21—O1	1.220 (3)
C7—C8	1.410 (3)	C21—O2	1.298 (3)
C8—C9	1.367 (3)	C22—O4	1.236 (3)
C8—H8B	0.93	C22—O3	1.279 (3)
C9—C10	1.399 (3)	C23—O6	1.198 (3)
C9—H9	0.93	C23—O5	1.334 (3)
C10—N2	1.323 (3)	C24—O7	1.200 (3)
C10—H10	0.93	C24—O8	1.320 (3)
C11—N2	1.354 (3)	N1—Cu1	2.0072 (17)
C11—C12	1.434 (3)	N2—Cu1	2.0119 (19)
C12—N1	1.356 (3)	O2—Cu1	1.9640 (15)
C13—C21	1.525 (3)	O1W—HW11	0.82 (3)
C13—C14	1.540 (3)	O1W—HW12	0.822 (19)
C13—C18	1.556 (3)	O3—Cu1^i^	1.9355 (15)
C13—H13	0.98	O5—H5	0.82
C14—C20	1.505 (3)	O7—Cu1^ii^	2.3398 (18)
C14—C15	1.554 (3)	O8—H8	0.82
C14—H14	0.98	Cu1—O3^iii^	1.9355 (15)
C15—C24	1.516 (3)	Cu1—O7^iv^	2.3398 (18)
			
N1—C1—C2	122.0 (2)	C15—C16—C17	108.75 (16)
N1—C1—H1	119.0	C23—C16—H16	105.8
C2—C1—H1	119.0	C15—C16—H16	105.8
C3—C2—C1	119.7 (2)	C17—C16—H16	105.8
C3—C2—H2	120.2	C19—C17—C16	111.41 (18)
C1—C2—H2	120.2	C19—C17—C18	106.48 (17)
C2—C3—C4	120.0 (2)	C16—C17—C18	105.53 (16)
C2—C3—H3	120.0	C19—C17—H17	111.1
C4—C3—H3	120.0	C16—C17—H17	111.1
C12—C4—C3	116.4 (2)	C18—C17—H17	111.1
C12—C4—C5	118.2 (2)	C22—C18—C13	116.14 (16)
C3—C4—C5	125.3 (2)	C22—C18—C17	108.15 (17)
C6—C5—C4	121.3 (2)	C13—C18—C17	106.20 (16)
C6—C5—H5A	119.4	C22—C18—H18	108.7
C4—C5—H5A	119.4	C13—C18—H18	108.7
C5—C6—C7	121.6 (2)	C17—C18—H18	108.7
C5—C6—H6	119.2	C20—C19—C17	114.4 (2)
C7—C6—H6	119.2	C20—C19—H19	122.8
C11—C7—C8	116.8 (2)	C17—C19—H19	122.8
C11—C7—C6	118.0 (2)	C19—C20—C14	113.8 (2)
C8—C7—C6	125.2 (2)	C19—C20—H20	123.1
C9—C8—C7	119.4 (2)	C14—C20—H20	123.1
C9—C8—H8B	120.3	O1—C21—O2	124.26 (19)
C7—C8—H8B	120.3	O1—C21—C13	121.4 (2)
C8—C9—C10	119.8 (2)	O2—C21—C13	114.15 (18)
C8—C9—H9	120.1	O4—C22—O3	124.92 (19)
C10—C9—H9	120.1	O4—C22—C18	121.8 (2)
N2—C10—C9	122.1 (2)	O3—C22—C18	113.28 (18)
N2—C10—H10	119.0	O6—C23—O5	124.8 (2)
C9—C10—H10	119.0	O6—C23—C16	125.9 (2)
N2—C11—C7	123.2 (2)	O5—C23—C16	109.01 (19)
N2—C11—C12	116.08 (19)	O7—C24—O8	122.7 (2)
C7—C11—C12	120.70 (19)	O7—C24—C15	123.8 (2)
N1—C12—C4	123.4 (2)	O8—C24—C15	113.40 (19)
N1—C12—C11	116.42 (17)	C1—N1—C12	118.41 (18)
C4—C12—C11	120.14 (19)	C1—N1—Cu1	128.81 (15)
C21—C13—C14	114.04 (17)	C12—N1—Cu1	112.72 (14)
C21—C13—C18	115.24 (16)	C10—N2—C11	118.6 (2)
C14—C13—C18	110.05 (17)	C10—N2—Cu1	128.54 (16)
C21—C13—H13	105.5	C11—N2—Cu1	112.83 (15)
C14—C13—H13	105.5	C21—O2—Cu1	125.43 (14)
C18—C13—H13	105.5	HW11—O1W—HW12	109 (4)
C20—C14—C13	111.19 (17)	C22—O3—Cu1^i^	114.82 (14)
C20—C14—C15	105.24 (17)	C23—O5—H5	109.5
C13—C14—C15	107.38 (17)	C24—O7—Cu1^ii^	130.37 (16)
C20—C14—H14	110.9	C24—O8—H8	109.5
C13—C14—H14	110.9	O3^iii^—Cu1—O2	89.24 (7)
C15—C14—H14	110.9	O3^iii^—Cu1—N1	162.98 (7)
C24—C15—C16	114.85 (17)	O2—Cu1—N1	94.97 (7)
C24—C15—C14	110.54 (18)	O3^iii^—Cu1—N2	96.53 (7)
C16—C15—C14	107.01 (16)	O2—Cu1—N2	170.11 (7)
C24—C15—H15	108.1	N1—Cu1—N2	81.84 (7)
C16—C15—H15	108.1	O3^iii^—Cu1—O7^iv^	92.84 (7)
C14—C15—H15	108.1	O2—Cu1—O7^iv^	84.71 (6)
C23—C16—C15	116.02 (18)	N1—Cu1—O7^iv^	103.96 (7)
C23—C16—C17	113.89 (18)	N2—Cu1—O7^iv^	86.97 (7)
			
N1—C1—C2—C3	0.4 (4)	C15—C14—C20—C19	57.3 (2)
C1—C2—C3—C4	0.2 (4)	C14—C13—C21—O1	−1.8 (3)
C2—C3—C4—C12	−0.3 (4)	C18—C13—C21—O1	−130.5 (2)
C2—C3—C4—C5	178.6 (2)	C14—C13—C21—O2	−177.45 (17)
C12—C4—C5—C6	−2.2 (4)	C18—C13—C21—O2	53.9 (2)
C3—C4—C5—C6	178.8 (3)	C13—C18—C22—O4	17.8 (3)
C4—C5—C6—C7	0.4 (4)	C17—C18—C22—O4	−101.4 (2)
C5—C6—C7—C11	2.0 (4)	C13—C18—C22—O3	−164.32 (18)
C5—C6—C7—C8	−176.8 (3)	C17—C18—C22—O3	76.5 (2)
C11—C7—C8—C9	0.9 (4)	C15—C16—C23—O6	−114.2 (3)
C6—C7—C8—C9	179.8 (2)	C17—C16—C23—O6	13.2 (3)
C7—C8—C9—C10	0.3 (4)	C15—C16—C23—O5	71.4 (2)
C8—C9—C10—N2	−0.4 (4)	C17—C16—C23—O5	−161.24 (19)
C8—C7—C11—N2	−2.3 (3)	C16—C15—C24—O7	−23.1 (3)
C6—C7—C11—N2	178.8 (2)	C14—C15—C24—O7	−144.3 (2)
C8—C7—C11—C12	176.3 (2)	C16—C15—C24—O8	160.03 (19)
C6—C7—C11—C12	−2.6 (3)	C14—C15—C24—O8	38.8 (2)
C3—C4—C12—N1	−0.1 (3)	C2—C1—N1—C12	−0.8 (4)
C5—C4—C12—N1	−179.1 (2)	C2—C1—N1—Cu1	−177.84 (18)
C3—C4—C12—C11	−179.3 (2)	C4—C12—N1—C1	0.6 (3)
C5—C4—C12—C11	1.6 (3)	C11—C12—N1—C1	179.9 (2)
N2—C11—C12—N1	0.2 (3)	C4—C12—N1—Cu1	178.13 (18)
C7—C11—C12—N1	−178.5 (2)	C11—C12—N1—Cu1	−2.6 (2)
N2—C11—C12—C4	179.5 (2)	C9—C10—N2—C11	−0.9 (4)
C7—C11—C12—C4	0.8 (3)	C9—C10—N2—Cu1	−179.39 (19)
C21—C13—C14—C20	−88.1 (2)	C7—C11—N2—C10	2.3 (3)
C18—C13—C14—C20	43.2 (2)	C12—C11—N2—C10	−176.4 (2)
C21—C13—C14—C15	157.29 (17)	C7—C11—N2—Cu1	−179.00 (18)
C18—C13—C14—C15	−71.42 (19)	C12—C11—N2—Cu1	2.3 (2)
C20—C14—C15—C24	56.4 (2)	O1—C21—O2—Cu1	−23.2 (3)
C13—C14—C15—C24	174.93 (17)	C13—C21—O2—Cu1	152.23 (14)
C20—C14—C15—C16	−69.3 (2)	O4—C22—O3—Cu1^i^	9.8 (3)
C13—C14—C15—C16	49.2 (2)	C18—C22—O3—Cu1^i^	−167.97 (14)
C24—C15—C16—C23	26.6 (3)	O8—C24—O7—Cu1^ii^	−7.8 (4)
C14—C15—C16—C23	149.70 (18)	C15—C24—O7—Cu1^ii^	175.59 (14)
C24—C15—C16—C17	−103.3 (2)	C21—O2—Cu1—O3^iii^	−77.88 (17)
C14—C15—C16—C17	19.8 (2)	C21—O2—Cu1—N1	85.60 (17)
C23—C16—C17—C19	−91.2 (2)	C21—O2—Cu1—O7^iv^	−170.81 (17)
C15—C16—C17—C19	39.8 (2)	C1—N1—Cu1—O3^iii^	94.5 (3)
C23—C16—C17—C18	153.60 (18)	C12—N1—Cu1—O3^iii^	−82.7 (3)
C15—C16—C17—C18	−75.3 (2)	C1—N1—Cu1—O2	−9.3 (2)
C21—C13—C18—C22	26.2 (3)	C12—N1—Cu1—O2	173.52 (15)
C14—C13—C18—C22	−104.5 (2)	C1—N1—Cu1—N2	−179.9 (2)
C21—C13—C18—C17	146.43 (17)	C12—N1—Cu1—N2	2.94 (15)
C14—C13—C18—C17	15.8 (2)	C1—N1—Cu1—O7^iv^	−95.1 (2)
C19—C17—C18—C22	59.7 (2)	C12—N1—Cu1—O7^iv^	87.74 (15)
C16—C17—C18—C22	178.16 (17)	C10—N2—Cu1—O3^iii^	−21.4 (2)
C19—C17—C18—C13	−65.7 (2)	C11—N2—Cu1—O3^iii^	160.06 (15)
C16—C17—C18—C13	52.9 (2)	C10—N2—Cu1—N1	175.7 (2)
C16—C17—C19—C20	−57.8 (2)	C11—N2—Cu1—N1	−2.86 (15)
C18—C17—C19—C20	56.8 (2)	C10—N2—Cu1—O7^iv^	71.1 (2)
C17—C19—C20—C14	5.7 (3)	C11—N2—Cu1—O7^iv^	−107.43 (15)
C13—C14—C20—C19	−58.6 (2)		

Symmetry codes: (i) *x*+1, *y*, *z*; (ii) −*x*+2, *y*+1/2, −*z*+2; (iii) *x*−1, *y*, *z*; (iv) −*x*+2, *y*−1/2, −*z*+2.

### Hydrogen-bond geometry (Å, °)

**Table:**

*D*—H···*A*	*D*—H	H···*A*	*D*···*A*	*D*—H···*A*
O5—H5···O1W	0.82	1.84	2.567 (2)	148
O8—H8···O2^ii^	0.82	1.79	2.594 (2)	166
O1W—HW11···O4^v^	0.82 (3)	1.97 (3)	2.777 (3)	167 (3)
O1W—HW12···O1^vi^	0.82 (2)	2.05 (3)	2.763 (3)	145 (4)

Symmetry codes: (ii) −*x*+2, *y*+1/2, −*z*+2; (v) *x*, *y*, *z*+1; (vi) *x*+1, *y*, *z*+1.
